# The beneficial effect of cold atmospheric plasma on parameters of molecules and cell function involved in wound healing in human osteoblast-like cells in vitro

**DOI:** 10.1007/s10266-020-00487-y

**Published:** 2020-02-06

**Authors:** B. Eggers, J. Marciniak, S. Memmert, F. J. Kramer, J. Deschner, M. Nokhbehsaim

**Affiliations:** 1 Department of Oral Surgery, Center of Dento-Maxillo-Facial Medicine, University of Bonn, Bonn, Germany; 2 Section of Experimental Dento-Maxillo-Facial Medicine, Center of Dento-Maxillo-Facial Medicine, University of Bonn, Bonn, Germany; 3 Department of Orthodontics, Center of Dento-Maxillo-Facial Medicine, University of Bonn, Bonn, Germany; 4 Department of Craniomaxillofacial Surgery, Center of Dento-Maxillo-Facial Medicine, University of Bonn, Bonn, Germany; 5 Department of Periodontology and Operative Dentistry, University Medical Center of the Johannes Gutenberg University, Mainz, Germany

**Keywords:** Cold atmospheric plasma, MG63 cells, Wound healing, Cell viability, Cell proliferation

## Abstract

The aim of this study was to analyse the effect of cold atmospheric plasma (CAP) on human osteoblast-like cells in vitro. Additionally, underlying intracellular mechanisms were to be studied. Human osteoblast-like (MG63) cells were exposed to CAP for 60 s. The effects of CAP on key molecules essential for the wound healing response were studied using real-time PCR, ELISA and immunocytochemistry. For studying intracellular signalling pathways, MAP kinase MEK 1/2 was blocked. Cell viability was analysed by an XTT assay and with an EVE automated cell counter. Cell migration was examined by an in vitro wound healing assay.

CAP exposition on osteoblast-like cells caused a significant upregulation of interleukin (IL)-1β, IL-6, IL-8, tumor necrosis factor (TNF)α, cyclooxygenase (COX)2, collagen (COL) 1α, matrix metalloproteinase (MMP)1, Ki67, proliferating-cell-nuclear-antigen (PCNA) and chemokine ligand (CCL)2 mRNA expression at 1 day. Interestingly, after blocking of MAP kinase, CAP-induced upregulation of Ki67 was inhibited by 57%. Moreover, CAP treatment improved significantly osteoblast-like cell viability as compared to untreated cells at 1 day. Beneficial effect of CAP treatment was shown by an in vitro wound healing assay, displaying a significant faster wound closure. Our findings provide evidence that CAP exposure effects gene and protein regulation in human osteoblast-like cells. Furthermore, CAP treatment has a positive impact on wound closure in an in vitro setting and might improve existing concepts of hard tissue regeneration in the future.

## Introduction

The healing post-operative process after oral surgery interventions include the repair and regeneration of soft and hard tissues [1–3]. In own previous studies it was demonstrated that cold atmospheric plasma (CAP) could positively influence periodontal wound healing by change of critical molecules at transcriptional level, increase of cell viability and wound closure rate in human periodontal ligament cells (hPDL) [4]. The healing of hard tissue is a major step for the entire regeneration of an affected area, forming its stabilizing scaffold. Bone tissue healing is a multifactorial process involving various cell types such as osteoblasts and osteoclasts as well as different immune cells [5, 6]. The regeneration process, which can be divided into different stages, is initiated by tissue damage, followed by a local immune reaction, which plays a significant role in the entire process of wound healing [7, 8]. During the inflammation process following the traumatic stimulus immediately a large number of mediators, e.g. factors such as IL-1β, IL-6, IL-8, CCL2 and TNFα are expressed [9–12]. Nevertheless compared to soft tissue repair reactions the inflammatory process is then downregulated in the early phase of injury, between 24 and 36 h [13]. Simultaneously to the first inflammation process high amounts of angiogenic factors promote revascularisation within the initial hematoma, which develops after the traumatic disruption of blood vessels. The organism responses by activating primary haemostasis to stop the bleeding but also to prevent infection.

Following bone healing different cytokines and growth factors produced by the osteoblasts promote the ossification process, such as COL1α [14, 15]. Within the first days of bone healing, markers of proliferation are expressed, such as PCNA or Ki67 [16, 17]. In the process of bone remodelling MMPs such as MMP1 play a central role. They catalyse the enzymatic remodelling of the extracellular matrix (ECM) [18]. More and more chondroid tissue fills the impaired area and starts to develop a soft callus, which supports the development of osteoblasts [19]. Collagenous tissue is produced by the osteoblasts, which promote its mineralisation by releasing calcium and phosphate containing matrix vesicles [20]. During the ossification process the osteoblasts immure themselves with hydroxyapatite and become osteocytes, forming the new bone within 3–6 months [14].

This bone regeneration process is not only confined to tissue damage: a special attribute of bone is its high potential of constant remodelling by periodic resorption and bone formation [21]. Especially the alveolar bone is characterized by quick bone remodelling caused by different dynamic actions, such as masticating, and undergoes resorption by loss of this stimulus [22, 23].

The recovery of stability of hard tissue defects is the main goal in the healing of hard tissue wounds. The regeneration process is influenced by different extrinsic or intrinsic factors such as personal physical constitution, systemic diseases or the consumption of nicotine or alcohol [24–26]. Additionally topic treatment with different growth factors or chemokines has been described to enhance wound healing [27–29].

Newly cold atmospheric plasma (CAP), a room temperate ionised gas, known as the fourth state of aggregation, has lately been identified to enhance wound healing [30]. It can be achieved by energizing gases like inert gases such as argon or by ionising the ambient air to create reactive components with multiple effects. Many authors have described the positive effect of CAP in accelerating wound healing, erasing bacteria or reducing candida [31–35]. Incidentally, the effect of CAP on critical cell functions is linked with active plasma components [36]. However, plasma research is a new field and the exact mode of action of CAP on the treated cells and tissue requires further investigation. Various effects of CAP on gene regulation have been observed in different cell types such as keratinocytes or gingival fibroblasts [37, 38]. Additionally, we have recently shown CAP effects on periodontal cells in vitro [4]. Apart from these soft tissue cells other resident oral cell types should be analysed. CAP effect on human bone cells and development is poorly understood. Therefore, the aim of the present study was to analyse CAP effects on hard tissue cells. Thus, as a primary human bone cell line, human osteoblast-like cells, MG63 cells, were used for a better understanding of underlying mechanism that modulate the bone cell response of CAP exposure.

## Materials and methods

### MG63 cell culture

Human osteoblast-like MG63 osteosarcoma cells (ATCC, CRL-1427™) (Sigma-Aldrich, Taufkirchen, Germany) were cultured in Dulbecco's modified essential medium (DMEM, Invitrogen, Dreieich, Germany) supplemented with 10% fetal bovine serum (FBS, Invitrogen), 100 units penicillin, and 100 μg/mL streptomycin (Invitrogen) at 37 °C in a humidified atmosphere of 5% CO_2_ and 95% humidity. After passaging, cells were seeded into 35 × 10 mm Petri dishes and cultured to 80% confluence. 1 day prior to the experiments, the FBS concentration was reduced to 1%. Medium was changed every 2 days throughout the whole cultivation period.

### Cold plasma application and preliminary experiments

Ambient air CAP was generated by the plasma ONE device (Plasma ONE MEDICAL, Plasma MEDICAL SYSTEMS® GmbH, Nievern, Germany), a volume dielectric barrier discharge (DBD). CAP is generated by a pulsed direct current (35 V), which is transformed to high voltage leading to an electric field at the tip of the probe to create a corona-discharge. The ambient air gases—mainly nitrogen, but also oxygen and argon, are converted into CAP: the CAP can be used at 5 levels of intensity, modulating the high voltage (3–18 kV). MG63 cells were exposed to CAP treatment for 60 s as previously described [4]. Optimal conditions were selected after preliminary experiments.

### Analysis of gene expression

24 h after CAP application total RNA of MG63 cells was extracted using an RNA extraction kit (Qiagen, Hilden, Germany). iScript™ Select cDNA Synthesis Kit (Bio-Rad Laboratories, Munich, Germany) was used to reversely transcribe 1 μg of RNA at 42 °C for 90 min followed by 85 °C for 5 min. 1 μl of cDNA was amplified as a template in a 25 μl reaction mixture containing 12.5 μl 2 × QuantiFast SYBR Green PCR Master Mix (Qiagen), 2.5 μl of primers (0.5 μM each), and 9 μl deionized water. The mixture was heated at 95 °C for 5 min initially, followed by 40 cycles with denaturation at 95 °C for 10 s and combined annealing/extension at 60 °C for 30 s. Glyceraldehyde 3-phosphate dehydrogenase (GAPDH) was used as an endogenous control. mRNA expression of IL-1β, IL-6, IL-8, TNFα, COX2, COL1α, MMP1, Ki67, PCNA and CCL2 was detected by real-time PCR using the iCycler iQ™ detection system (Bio-Rad Laboratories, Hercules, CA, USA), SYBR Green (Bio-Rad Laboratories), and specific primers (QuantiTect Primer Assay, Qiagen). mRNA was quantified by the comparative threshold cycle method.

### Inhibition of specific signaling pathway

To unravel intracellular signaling pathways underlying the regulatory CAP effect on cell proliferation cells were pre-treated with a specific inhibitor of mitogen-activated protein kinase pathway, MEK1/2 (U0126; 10 μM; Calbiochem, San Diego, CA, USA) 60 min prior to CAP exposure, in an additional experimental set. Next, gene regulation was analysed by real-time PCR.

### ELISA assay

The enzyme-linked immunosorbent assay (ELISA) as an antibody-based method was used for quantifying protein level, in MG63 cell culture supernatants by commercially available kits according to the manufacturer’s instructions. After CAP treatment of MG63 cells the protein levels of MMP1 (ELH-MMP1-1; RayBiotech, USA), and CCL2 (DCP00; R&D Systems, Wiesbaden, Germany) were evaluated after 1 d. The microplates were coated with a specific antibody directed against the protein to be detected. A microplate reader (PowerWave x, BioTek Instruments, Winooski, VT, USA) at 450 nm was used to analyse the absorbance. Data were normalized by total protein concentration using Pierce protein BCA Assay Kit (Thermo Scientific, Pierce Biotechnology, Rockford, USA).

### Immunocytochemistry staining

MG63 cells were seeded on Thermanox 13 mm Nunc™ glass coverslips (Thermo Fisher Scientific Inc., Schwerte, Germany) until reached 70% confluence. CAP treated cells were incubated for 24 h. For the staining, cells were at first fixed in 4% paraformaldehyde (Sigma-Aldrich) at pH 7.4 and room temperature for 10 min, washed with PBS (Sigma-Aldrich), and treated with 0.1% Triton X-100 (Sigma-Aldrich) for 5 min. Serum block (Dako, Hamburg Germany) was used for 20 min to reduce background staining. MG63 cells were washed with PBS (Sigma-Aldrich) and then incubated with a rabbit polyclonal Anti-COL1 antibody (Abcam, Cambridge, UK) 1:200 at 4 °C overnight. Antibody goat anti-rabbit IgG HRP (Dako) was used as a secondary antibody for 45 min. Finally, DAB chromogen staining (Thermo Fisher Scientific) was applied for 10 min at room temperature to visualize antibody binding. Two washing steps with PBS (Sigma-Aldrich) were performed between each incubation step. Results were analysed using the Axioskop 2 microscope (Carl Zeiss, Jena, Germany) with an AxioCam MRc camera (Carl Zeiss) and the AxioVision 4.7 software (Carl Zeiss).

### Wound healing assay

The MG63 cell monolayers were wounded with 3 mm wide defects (wounds) in a standardized manner and treated with CAP. Wound closure was evaluated in monolayers even untreated or treated with CAP over a period of 72 h using JuLI™ Br Live Cell Analyzer and the JuLI™ Br PC software (both: NanoEnTek, Seoul, Korea). The device analysed automatically the cell repopulation of the wounded area every 30 min. Subsequently, the wound closure was calculated by dividing the repopulated area through the cell free area created immediately after wounding.

### Quantitating and viability testing of cells

A commercial cell proliferation Assay XTT (Applichem, Darmstadt, Germany) was used to determine the number of viable cells. MG63 cells were seeded into 96-well plates at 5 × 10^3^ cells/well and grown to 80% confluence. CAP was applied on cell monolayers as described before. After 24 h of cultivation XTT reagent solution was added to the medium for 4 h. Results were evaluated using a microplate reader (PowerWave x, BioTek Instruments, Winooski, VT, USA) with a measurement of absorbance at 475 nm with correction wavelength 630–690 nm. Additionally, cell number was determined by an Automated Cell Counter, EVE™ (NanoEnTek Inc, Seoul, Korea) immediately before and 24 h after CAP application. Untreated cells were used as control.

### Statistical analysis

All experiments were performed in triplicates and repeated at least twice. Mean values and standard errors of the mean (SEM) were calculated. Parametric (ANOVA) and nonparametric (Mann–Whitney *U*) tests were applied for statistical analysis using the GraphPad Prism Software (GraphPad Software, San Diego, USA) at *p* < 0.05.

### Artwork

Graphics software CorelDRAW X6 (Corel Corporation, Ottawa, Canada) was used to create the artwork.

## Results

### Effect of CAP treatment on proinflammatory mediators

**Fig. 1:**
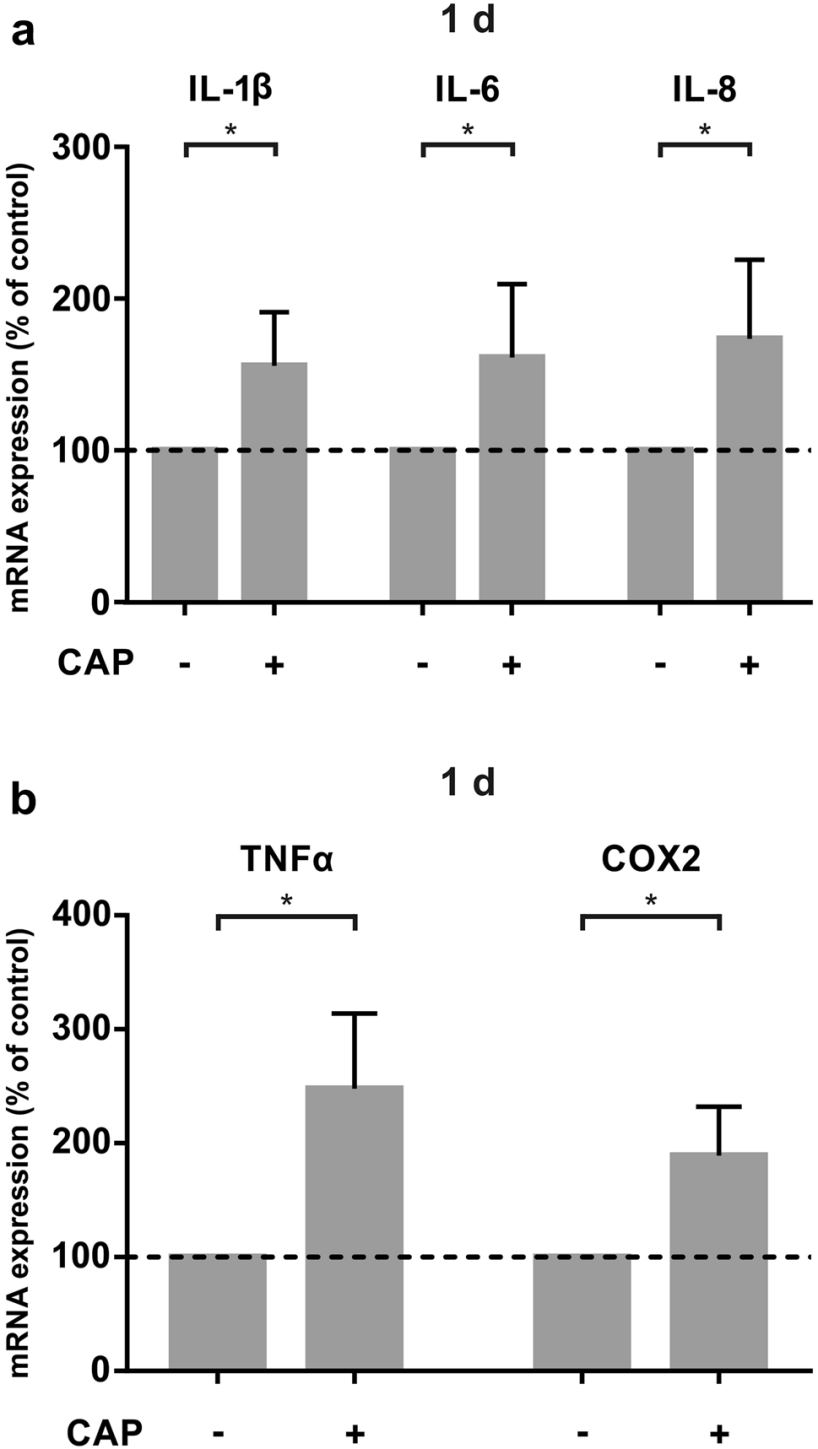
mRNA-expression of proinflammatory markers in MG63 cells after 60 s of CAP treatment at 1 d ( +) as compared to untreated cells (−). **a** mRNA-Expression of IL-1β, IL-6 and IL-8, (*n* = 12). **b** mRNA-expression of TNFα, and COX2, (*n* = 12). *Statistical significance

The application of CAP on MG63 cells significantly increased the mRNA expression of proinflammatory cytokines IL-1β, IL-6, and chemotactic factor IL-8 after 1 day compared to untreated cells (Fig. 1a). Additionally, we observed a significant upregulation of mRNA expression of TNFα and COX2 after CAP application, both being well known as important genes for primary inflammation phase concerning wound healing (Fig. 1b).

### Effect of CAP treatment on extracellular matrix regulation

**Fig. 2:**
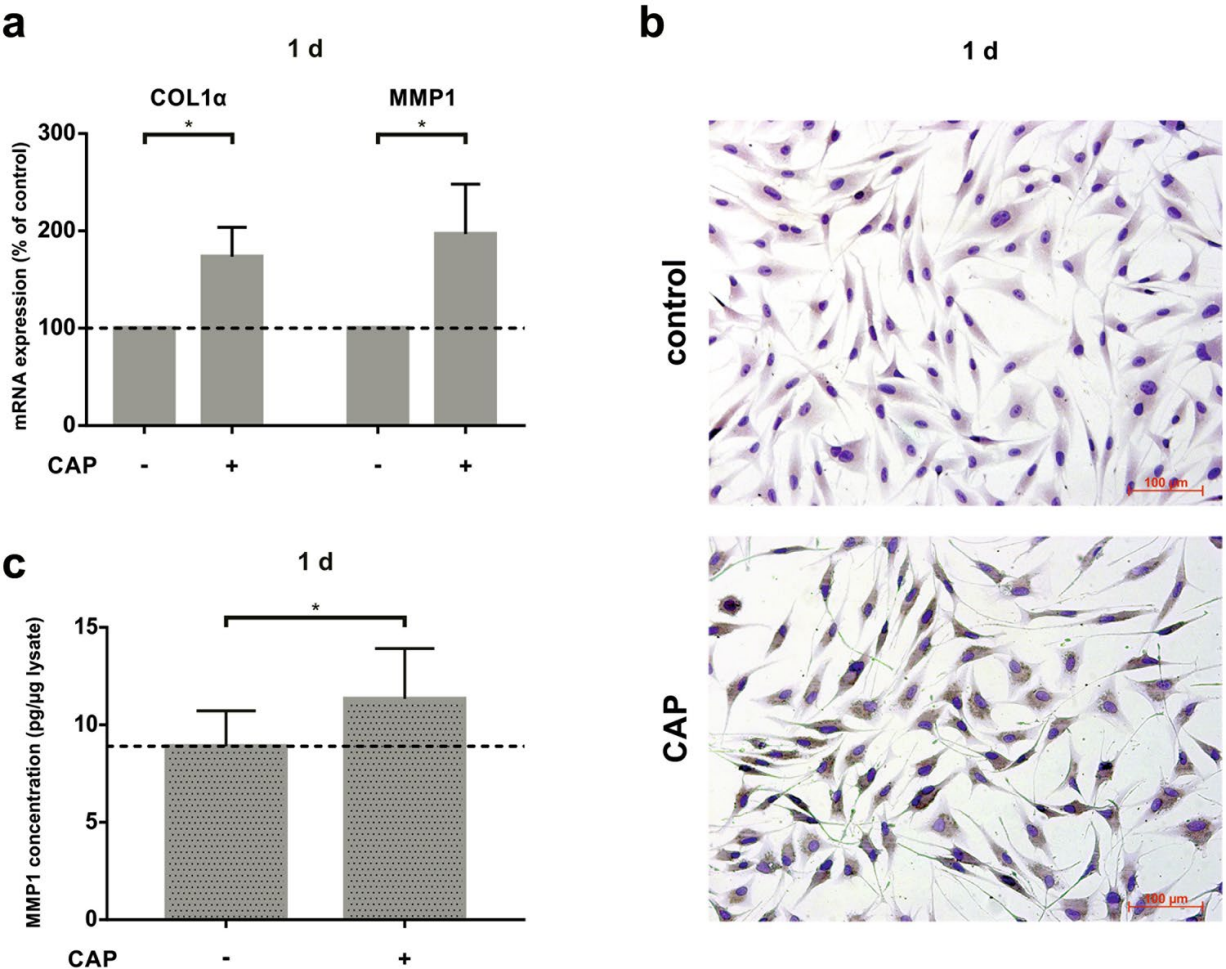
Stimulatory effect of CAP on ECM component in MG63 cells after 60 s of CAP treatment at 1 day ( +) as compared to untreated cells (−). **a** mRNA-Expression of COL1α and MMP1, (*n* = 12). **b** Protein synthesis of COL1 shown in a representative experiment by ICC staining. **c** Protein synthesis of MMP1 in MG63 cell culture supernatants shown by ELISA, (*n* = 6). *Statistical significance

Additionally, we studied COL1α expression as an important wound healing component for extracellular matrix (ECM) synthesis. COL1α mRNA was upregulated 1 d after CAP treatment (Fig. 2a). The high protein synthesis of COL1 could also been shown by immunocytochemistry in CAP treated cells after 1 d as compare to untreated MG63 cells (Fig. 2b). Since the hard tissue remodelling process is particularly associated with matrix metalloproteinases, we also analysed MMP1 gene expression after CAP treatment. Similar to COL1α, CAP treatment led to significant MMP1 mRNA expression after 1 day (Fig. 2a). To display its specific protein synthesis, we measured levels of MMP1 in MG63 cell culture supernatants by ELISA. 60 s of CAP treatment led to an increase of MMP1 protein level by about 34% after 1 day (Fig. 2c).

### Effect of CAP treatment on cell proliferation, number, viability and analysis of the molecular mechanism involved

**Fig. 3:**
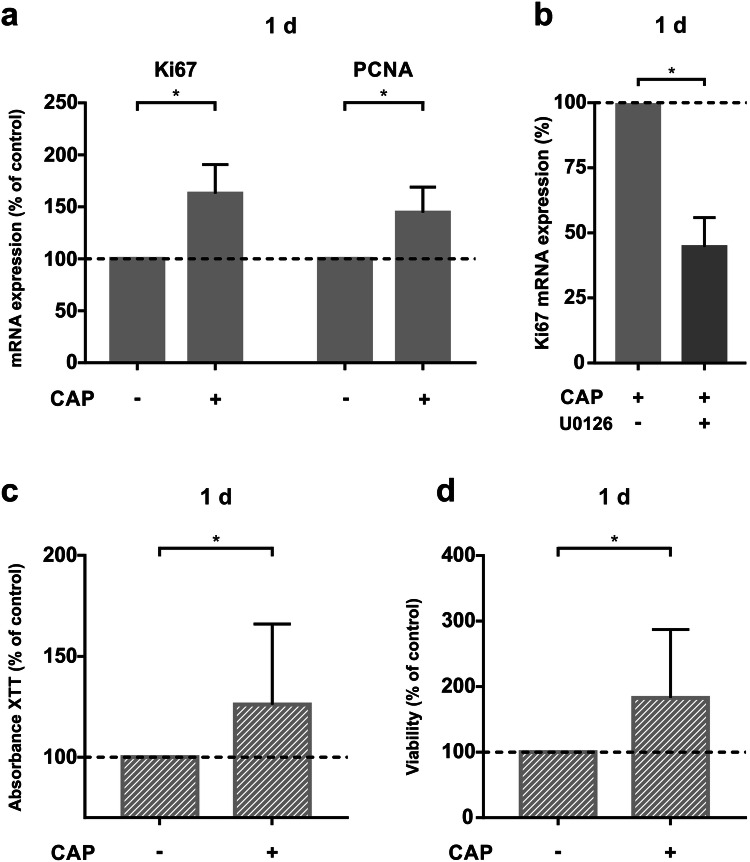
Effect of 60 s of CAP on MG63 cell proliferation, number, and viability at 1 d ( +) as compared to untreated cells (−). **a** mRNA-Expression of Ki67 and PCNA, (*n* = 12). **b** mRNA expression of Ki67 in MG63 cells after preincubation with MEK 1/2 inhibitor U0126, (*n* = 9). *Statistical significance. **c** XTT (cell proliferation-assay), (*n* = 18). **d** Viability of cells, determined by EVE automated cell counter, (*n* = 18). *Statistical significance

Due to the importance of cell proliferation for hard tissue wound healing, we studied the effect of CAP on specific cellular markers for proliferation genes, Ki67 and PCNA. 1 day after 60 s of CAP treatment, the mRNA expression of these genes was upregulated significantly compared to the control group (Fig. 3a). To explain underlying intracellular mechanisms involved in CAP-induced cell proliferation and thereby the accelerated wound closure, we used a MEK1/2 inhibitor. Pre-incubation of 60 min prior to CAP exposition was done to switch off intracellular MEK1/2 pathway. The CAP-induced upregulation of Ki67 was significantly reduced by about 57% due to the U0126 inhibitor (Fig. 3b). Furthermore, to visualize the cell proliferation according to CAP exposition we used a commercial XTT assay. 60 s CAP treatment of MG63 cells led to significantly enhanced cell viability after 1 day as compared to untreated cells (Fig. 3c). Similar results could be measured using an EVE™ automated cell counter concerning a higher number of viable cells (Fig. 3d).

### Effect of CAP treatment on chemotactic activity

**Fig. 4:**
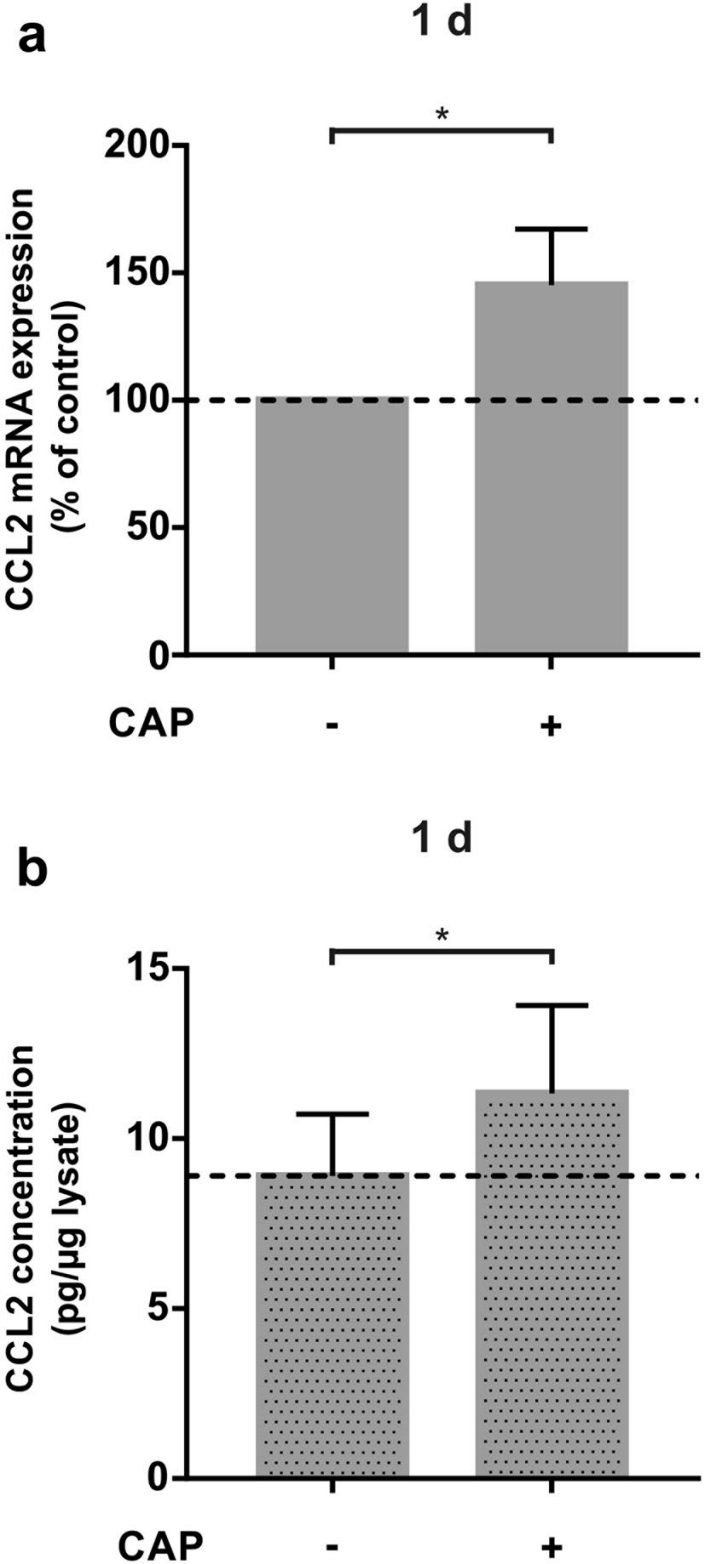
Effect of CAP treatment on chemotactic activity of MG63 cells after 60 s of CAP exposition on MG63 cells at 1 day ( +) as compared to untreated cells (−). **a** mRNA-Expression of CCL2, (*n* = 12). **b** Protein synthesis of CCL2 shown in cell culture supernatants shown by ELISA, (*n* = 12). *Statistical significance

We also studied CCL2 mRNA expression, which is known to be involved in immunoregulatory and inflammatory processes and plays an important part in the primary inflammation phase of wound healing (Fig. 4a). The CAP induced upregulation of CCL2 gene expression. The stimulatory effect of CAP could also be detected at the protein level of CCL2, which was significantly enhanced by about 27% as compared to the control group (Fig. 4b).

### Effects of CAP treatment on wound healing

**Fig. 5:**
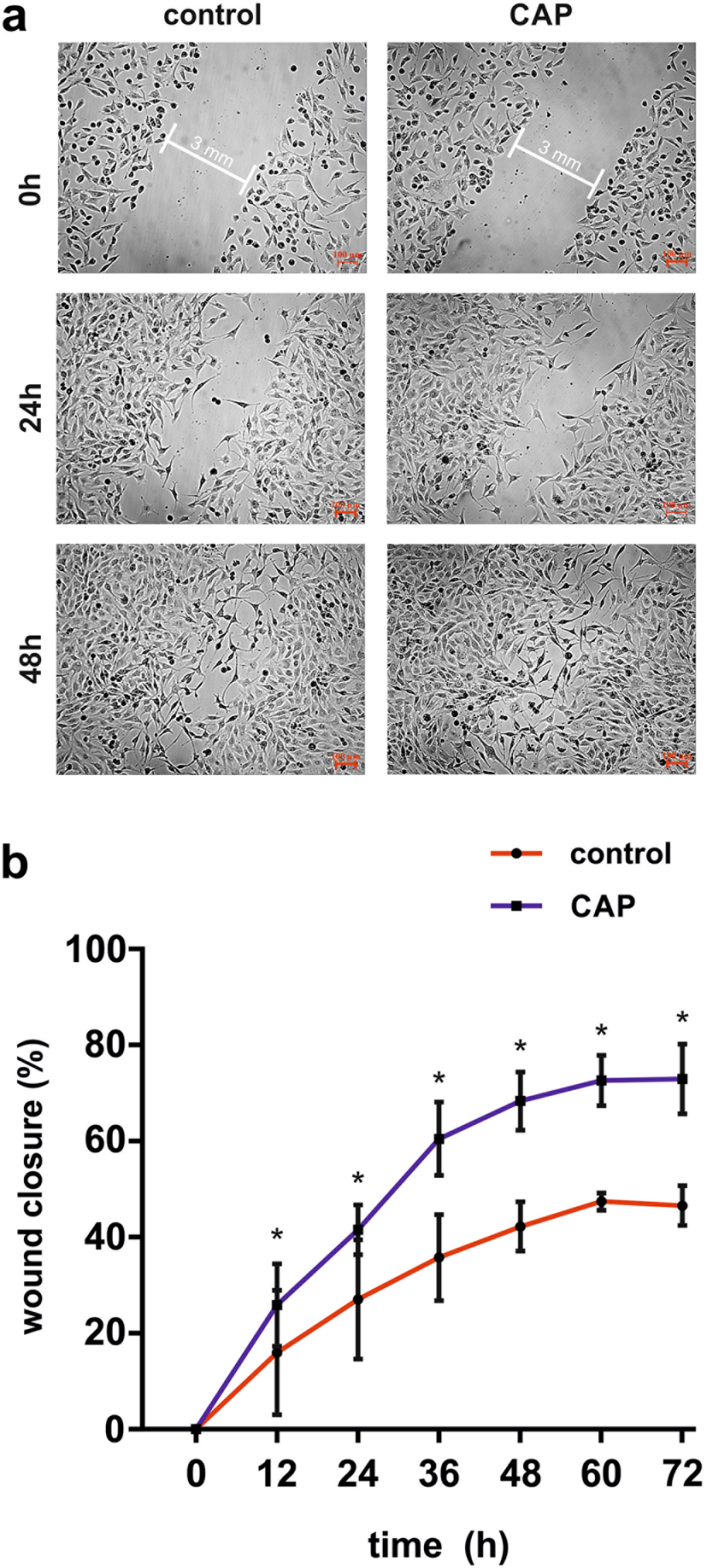
Stimulatory effect of CAP on wound healing in MG63 cells after CAP exposition for 60 s. **a** Microscopic imaging of wound-closure of wounded MG63 cell culture over a period of 72 h, (*n* = 3). **b** Graphical representation of wound-closure of wounded MG63 cell culture, (*n* = 3)

To confirm our previous results a live microscopy wound healing assay was used. CAP treated cells showed a significant faster closure of artificially induced wounds as compared to the non-plasma-treated group after 72 h (Fig. 5a, b).

## Discussion

This in vitro study demonstrated the beneficial effect of short time (60 s) application of CAP on parameters of molecules and cell function involved in wound healing in MG63 cells. It has been shown that inflammatory and proteolytic molecules in wound healing were upregulated after CAP application. Additionally, a CAP-mediated increase of proliferation, viability and wound closure of MG63 cell cultures has been displayed. These data suggest, that the application of CAP in critical settings might be beneficial for the promotion of wound healing processes.

In this study ambient air generated plasma was used to create CAP. In the literature also different methods of CAP generation are described: Inert gases such as argon or helium, but also nitrogen are more frequently chosen due to an easily achievable consistent and reproducible plasma quality, while ambient air requires the control of environmental conditions to obtain a similar result [39–43]. Nevertheless, the application of ambient air generated CAP is easier to manage with a small equipment for clinical application [44, 45]. Whether there are also differences in CAP effects has not finally been clarified yet. Daeschlein et al. at least observed similar effects in antimicrobial capacity of ambient air and argon generated CAP [46]. Further studies are required to investigate systematically possible differences between the effects of the different plasma sources on human cells.

The CAP application on MG63 cells led to a significant upregulation of genes of inflammation IL-1β, IL-6, and IL-8, TNFα, COX2 and CCL2 after 1 day. These mediators play an important role in wound healing managing the primary inflammation process and the organisation of the ECM, which are both crucial for the entire wound regulation [10, 47–49]. Especially CCL2 seems to be an important factor for bone remodelling [50]. The upregulating effect on these genes by CAP treatment gives evidence about higher levels of tissue regeneration. An upregulation of inflammatory genes CCL2, IL-6 and IL-8, 1 day after 2 min of argon-generated CAP exposition was also shown by Arndt et al. in dermal fibroblasts [51]. The CAP induced overexpression of inflammatory marker could be also demonstrated in argon-generated CAP exposed keratinocytes for IL-8 and in CAP exposed fibroblasts [52]. In a previous study an upregulation of TNFα, IL-1β and COX2 after time dependent ambient air-generated CAP exposition was seen after 1 day in human periodontal ligament cells [4]. Therefore, our study is an agreement with the finding by other investigators and further complements the current knowledge of CAP on critical cells of wound healing.

Apart from targeting inflammatory genes CAP exposition of MG63 cells also led to an upregulation of MMP1, an important regulator in tissue remodeling and cell–matrix regulation during wound healing [53]. These findings, as studied by ELISA, showed the stimulating effect of CAP concerning hard tissue regeneration.

The ECM is mainly composed of COL1α, whose production promotes tissue building and remodeling. Other markers for tissue remodeling are Ki67 and PCNA, which are expressed during cell proliferation. After CAP treatment of MG63 cells COL1α was upregulated significantly, which was also shown by immunocytochemistry regarding protein expression. The improved wound fill rate, which involves also cell migration, might be also because of the CAP-mediated improved collagen syntheses shown in the current study.

Similar findings concerning an elevated gene expression after CAP treatment were also observed in gingival fibroblasts and periodontal ligament cells [4, 38]. CAP treatment of MG63 cells also significantly upregulated Ki67 and PCNA, indicating a process of active cell proliferation. Similar effects on Ki67 expression were also observed in oral keratinocytes regarding argon generated CAP [37].

The increased level of cell proliferation after CAP treatment of MG63 cells was additionally confirmed by an XTT-proliferation assay. After CAP exposition osteoblast-like cells showed a significantly higher cell viability after 1 day. In addition, one could see an increased number of cells by cell counting after CAP treatment as compared to the control group. Similar results for the XTT assay were also observed in keratinocytes and fibroblasts after 24 h of argon-generated CAP exposition; but it seems that viability of keratinocytes increases up to 72 h after CAP treatment, whereas viability of fibroblasts decreases [54]. This viability loss of fibroblasts 24 h after 60 s of nitrogen-generated CAP treatment was also confirmed by Lee et al. [43]. There seem to be differences in cell lines regarding influence of CAP and influence of CAP activated components on cell viability. Further experiments will have to show the diverging effects of CAP treatment over time.

Corresponding to the increased level of cell proliferation, an increased wound closure in the in vitro scratch assay after 60 s of CAP treatment of MG63 cells was detected. A higher migration of cells after CAP treatment was also observed by other authors in fibroblasts and epithelial cells using argon as supplementary gas for the plasma [51, 55, 56]. Additionally, in a previous study we found similar effects for ambient air-generated CAP in periodontal ligament cells [4]. In contrast to these findings, experiments performed by Arndt et al. did not show any effect of CAP on migration of keratinocytes with argon as working gas [57]. Lendeckel and coworkers observed that argon-generated CAP application longer than 120 s seems to repress cell migration or may have a lethal effect on epithelial cells [55].

Interestingly, recent studies have shown a lethal effect of CAP on different kinds of cancer cells, such as melanoma, leukaemia or glioblastoma cells has been subject of various studies concerning inert gas generated plasma [58–60]. However, in the present examination MG63 osteosarcoma cells were used as osteoblast-like cells with different effects concerning CAP treatment. A reason for varying results concerning anti-cancer-capacity could be the different mode of CAP generation: We used ambient-air generated CAP instead of argon-generated CAP. Another reason could be a difference of CAP-related effect on various cell types. To the best of our knowledge, this observation of CAP effects on MG63 cells is the first in literature. Further analysis of osteoblasts could help to better understand underlying mechanisms.

All together the CAP related effects might be dependent from different settings, for example a difference in working gas, intensity, distance, duration or also specific cell response. Future investigations should help to clearly understand the optimal conditions for the individual type of CAP application.

Furthermore, CAP treatment of different scaffolds seems also to be an interesting field of future research in regenerative medicine: Thus CAP treatment of nanofibers led to an improved attachment of cells and proliferation of fibroblasts and osteoblasts [61]. Zhu et al. showed an increase of hydrophilic attributes of poly caprolactone scaffolds after CAP treatment [62]. A combination of CAP treated wounds and tissue engineering might improve existing concepts of hard tissue regeneration.

Finally, animal models and clinical trials will have to confirm the present results and help to clarify the parameters of CAP treatment and its effect on different cell types, facilitating to implement CAP treatment as promising therapeutic options in medicine.

In summary, our in vitro study showed that short time application of CAP had beneficial effects on osteoblast-like cells with regard to the expression of molecules critically involved in wound healing. Additionally, CAP has stimulatory effect on cell proliferation, viability and in vitro wound closure. Therefore, the present findings suggest that the clinical application of CAP might be beneficial and may even open up a new treatment strategy in oral surgery. CAP could enhance healing after both complex interventions, such as cystectomies or surgical removal of impacted third molars and less invasive surgical procedures such as regular tooth extractions. Especially patients with an impaired wound healing (e.g. diabetics) might benefit from a better tissue recovery caused by CAP.
